# Vascularization mediated by mesenchymal stem cells from bone marrow and adipose tissue: a comparison

**DOI:** 10.1186/s13619-015-0025-8

**Published:** 2015-10-23

**Authors:** Karoline Pill, Sandra Hofmann, Heinz Redl, Wolfgang Holnthoner

**Affiliations:** 1 Ludwig Boltzmann Institute for Experimental and Clinical Traumatology, Donaueschingenstrasse 13, 1200 Vienna Austria; 2 Austrian Cluster for Tissue Regeneration, Vienna, Austria; 3 Department of Biomedical Engineering, Eindhoven University of Technology, 5600 MB Eindhoven, The Netherlands; 4 Institute for Complex Molecular Systems, Eindhoven University of Technology, PO Box 513, 5600 MB Eindhoven, The Netherlands; 5 Institute for Biomechanics, Swiss Federal Institute of Technology Zürich, Vladimir-Prelog-Weg 3, 8093 Zürich, Switzerland

**Keywords:** Vascularization, Endothelial cells, Mesenchymal stem cells, Co-culture, Molecular mechanisms

## Abstract

Tissue-engineered constructs are promising to overcome shortage of organ donors and to reconstruct at least parts of injured or diseased tissues or organs. However, oxygen and nutrient supply are limiting factors in many tissues, especially after implantation into the host. Therefore, the development of a vascular system prior to implantation appears crucial. To develop a functional vascular system, different cell types that interact with each other need to be co-cultured to simulate a physiological environment in vitro. This review provides an overview and a comparison of the current knowledge of co-cultures of human endothelial cells (ECs) with human adipose tissue-derived stem/stromal cells (ASCs) or bone marrow-mesenchymal stem cells (BMSCs) in three dimensional (3D) hydrogel matrices. Mesenchymal stem cells (MSCs), BMSCs or ASCs, have been shown to enhance vascular tube formation of ECs and to provide a stabilizing function in addition to growth factor delivery and permeability control for ECs. Although phenotypically similar, MSCs from different tissues promote tubulogenesis through distinct mechanisms. In this report, we describe differences and similarities regarding molecular interactions in order to investigate which of these two cell types displays more favorable characteristics to be used in clinical applications. Our comparative study shows that ASCs as well as BMSCs are both promising cell types to induce vascularization with ECs in vitro and consequently are promising candidates to support in vivo vascularization.

## Introduction

One of the major challenges in tissue engineering today is the realization of an integrated vascular network to provide adequate blood supply for living cells in tissue constructs. Limited by oxygen diffusion only, tissue-engineered products for skin- and cartilage-regeneration are already used in clinics [1]. However, organs with a more complex structure need a vascular system which integrates with the host vascular system to provide sufficient oxygen and nutrient supply to the cells [2].

To overcome the problem of missing ingrowth of host vessels into the construct, different approaches have been investigated [3]. Integration of signaling molecules in scaffolds to stimulate the growth of blood vessels from the host after in vivo implantation is a currently pursued strategy. Another method represents the in vitro generation of pre-vascularized tissues that will be connected to the host upon implantation. In the latter case, critical parameters for vascularization are the choice of cells, in addition to the culture medium, appropriate matrix, and cell seeding parameters [4–8].

Physiological microvasculature compromises endothelial cells (ECs) and in addition supportive cells, termed mural cells, to ensure controlled permeability, contraction, and stability and to supply growth factors. Mesenchymal stem cells (MSCs), a population of adult stem cells, develop into mural cells in vitro when co-cultured with ECs [9]. MSCs present a heterogeneous population of cells found in various tissues. Adipose tissue-derived stem/stromal cells (ASCs) [10] and bone marrow derived stem cells (BMSCs) provide autologous sources for adult stem cells. While both cell types are phenotypically very similar, they promote vascular tube formation via distinct molecular interactions [11]. These tube formations have been shown being capable of anastomosing with the host vascular system when implanted in vivo, independent of the MSC type used to induce EC outgrowth [12–15]. In this review, we focus on the differences and similarities in molecular interactions between human ECs on one side and on the other side human ASCs compared to human BMSCs in microvascular tube formation. Current knowledge is summarized and questions and discrepancies are critically discussed in order to investigate which of both types of MSCs might be more favorable in future clinical applications.

### Endothelial cells (ECs) for vascularization

The endothelium forms the inner cellular lining of blood and lymphatic vessels. Consequently, vascular ECs play a critical role in many physiological processes, including the control of vasomotor tone, blood cell trafficking, the maintenance of blood fluidity, hemostatic balance, permeability, angiogenesis, and both innate and adaptive immunity. They are also involved in vasculogenesis and angiogenesis during wound healing and repair and are therefore very important for vascularization strategies in tissue [6, 16–18]. Because of the remarkable heterogeneity of ECs in structure and function, defining the endothelium is difficult. Each vascular bed has its own specialized ECs which demonstrate unique structural (e.g., presence of Weibel-Palade bodies and morphology [19]) and functional properties, developmental programs, and roles in pathophysiology. Therefore, even after several attempts, there is a lack of a uniform cellular definition or functional characterization for this cell type [16–18]. Characteristic EC markers include CD31, vascular endothelial—cadherin (VE-cadherin), von Willebrand factor (vWF), vascular endothelial growth factor receptor 2 (VEGFR-2), thrombomodulin, and endoglin [20–25]. The co-existence of these markers on ECs is crucial in the definition of “EC” because most of these markers are not exclusively expressed on ECs. For prevascularizing strategies, ECs from different sources have been described. Human umbilical vein endothelial cells (HUVECs) are the most prominent endothelial cell type used as representatives of ECs in co-culture systems for vascularization because they are easy to isolate. While HUVECs are a population of ECs present in large blood vessels, ECs isolated from dermal blood and lymphatic vessels reflect molecular and morphological characteristics of the microcapillary bed. These microvascular ECs are found in the dermis of juvenile foreskin and different locations in adult skin, which are predominantly involved in tumor angiogenesis, wound healing, and inflammation and are named human dermal microvascular endothelial cells (HDMVECs) [26]. However, HUVECs and HDMVECs cannot be generally harvested from every individual and therefore do not provide an autologous cell source. A widely approved concept involves the existence of endothelial progenitor cells (EPCs) which were first described by Asahara et al. [27]. EPCs are human blood-circulating cells which are defined to be positive for CD133, CD34, and VEGFR-2. They were reported to differentiate ex vivo in cells with endothelial-like characteristics, including the formation of vessel-like structures under defined conditions and of cobblestone patterns when the population is growing to confluence in culture. Therefore, EPCs are primarily described by their cell-surface antigens and not like other progenitor cells by their ability to proliferate and to give rise to functional progeny [2, 6]. About 0.01 % of peripheral blood mononuclear cells (PBMNCs) are considered to be EPCs [28]. Today, there is no uniform clonogenic definition of EPCs or an experimental method to distinguish between different EPC subgroups [2, 6]. “Early” EPCs have been described as cells derived from bone marrow and expressing surface markers such as CD14, CD45, and CD133, which are shared with hematopoietic stem cell populations. They are thought to act in a paracrine manner, providing angiogenic factors [2, 29]. In contrast, “late” EPCs, also called outgrowth endothelial cells (OECs), appear much later in culture and exhibit characteristics which are reported as “typically endothelial” [2], meaning the expression of vWF, CD31, VE-cadherin, and connexin (Cx)43 and Cx45. They show high proliferative potential and are furthermore incorporated into resident vasculature [2, 29].

### Mesenchymal stem cells (MSCs) as source for vasculogenic growth factors

MSCs represent a resident population of stem cells in many adult tissues. Adult stem cells are undifferentiated cells capable of differentiation into specialized cell types of various tissues and avoid ethical concerns that arise with embryonic stem cells [30]. Today, there are many different approaches to characterize MSCs. The “Mesenchymal and Tissue Stem Cell Committee of the International Society for Cellular Therapy” proposed minimal criteria to define human MSCs. These include the following: (1) plastic-adherence of MSCs when maintained in standard culture conditions; (2) positive display for endoglin, CD73, and CD90 and negative for CD45, CD34, CD14 or CD11, CD97alpha or CD19, and HLA-DR surface molecules; and (3) differentiation potential into osteoblasts, adipocytes, and chondroblasts in vitro [11]. MSCs have the ability to differentiate into mural cells (pericytes or smooth muscle cells) in vitro. The differentiation occurs due to heterocellular communication via gap junctions of ECs and MSCs [31]. Mural cells make up the surrounding layer of blood vessels and are therefore crucial for conferring support and stabilization of these vessels [32]. There is evidence that MSCs express angiogenic factors like vascular endothelial growth factor (VEGF), fibroblast growth factor-2 (FGF-2), angiopoietin 1 (Ang-1), and epidermal growth factors (EGF) [33, 34]. Within the human body, the most common source for MSCs used in research today is bone marrow (BM). MSCs derived from bone marrow are referring to a heterogeneous cell population named BMSCs or just “MSCs”. However, BM aspiration is an invasive procedure and therefore other sources of MSCs have been under investigation. Another autologous source of MSCs beside BM is adipose tissue. Even small amounts of human subcutaneous adipose tissue contain enough multipotent MSCs to be cultured and expanded in vitro. Therefore, autologous cells can be harvested with minimal invasiveness, which makes fat a very attractive source for MSCs [35]. Remarkable heterogeneity of human ASCs has been reported due to different harvest, characterization, and culture techniques next to donor variations [36]. Standardized protocols which were proven to be reproducible would meet this problem have been published [10].

### Co-culture of endothelial cells (ECs) with bone marrow-mesenchymal stem cells (MSCs) 

**Fig. 1:**
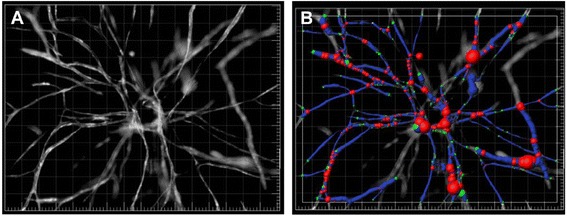
Example of how BMSCs support EC network formation in a fibrin matrix. **a** Fluorescent image of a capillary network on day 7. Thresholds were set before applying the Angiogenesis Tube Formation Application Module in Metamorph imaging software. **b** To visualize networks, the filament tracing function was used using Imaris. ECs are depicted in *blue* and BMSCs in *red* [39]

**Fig. 2:**
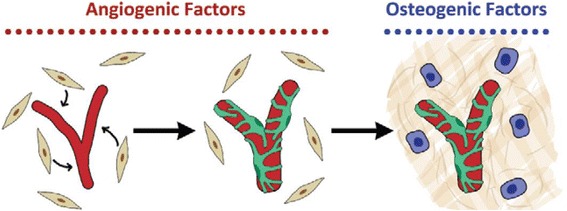
BMSCs supporting vascularization and forming mineralized bone matrix in a single co-culture system with HUVECs. When cultured in medium supported with angiogenic factors, BMSCs (*beige cells*) support ECs networks (*red*) as pericytes. When the co-culture system is supported with osteogenic factors, BMSCs undergo osteogenic differentiation (*blue cells*) and produce mineralized bone matrix (*beige lines*) [44]

Molecular interactions in co-cultures of BMSCs and ECs are of great interest because BMSCs promote the formation of vessel-like structures in three dimensional (3D) matrixes (Fig. 1), [9, 37–39] which is supported by the fact that HUVECs cultured without another cell type did not form any organized structures. After 14 days of co-culturing HUVECs with BMSCs, the expression of alpha smooth muscle actin (α-SMA) in BMSCs increased compared to expression before co-culture and surrounded EC cord-like structures. A multiplex chemiluminescent ELISA was performed by Verseijden et al. after 14 days of BMSC monoculture to analyze which angiogenic factors were increased in BMSC-conditioned medium compared to unconditioned medium or HUVEC-conditioned medium [9]. Results showed significantly higher amounts of hepatocyte growth factor (HGF), tissue inhibitor of metalloproteinase 1, and tissue inhibitor of metalloproteinase 2 (TIMP1 and TIMP2) in co-culture compared to monoculture. These factors are known also to regulate vessel formation. Additionally, HGF stimulates EC proliferation and induces vessel formation [40]. On the other hand, experiments using different concentrations of HGF failed to induce HUVEC outgrowth in monocultures. Regarding the high amount of TIMPs, further investigations have been performed because TIMPs had also been reported to inhibit the activity of matrix metalloproteinases (MMPs) [41]. Because of the high concentration of serum in the growth media, which is known to have a high MMP concentration itself, it was not possible to discriminate the amount of MMPs secreted specifically by BMSCs. Nevertheless, much of the fibrin was removed within the fibrin matrix, suggesting that MMPs or other factors were secreted in sufficient amount to degrade the fibrin. Despite the findings of angiogenesis related factors in BMSC-conditioned medium, HUVECs treated with this medium showed no positive effect regarding outgrowth or organization. The fact that BMSCs co-cultured with HUVECs induced outgrowth showed that HUVEC outgrowth and organization into vessel-like structures is not merely achieved by secreted factors. This suggests that direct cell-cell contact and reciprocal signaling may play an important role for ECs to form pre-vascular-like structures [9]. To investigate the angiogenesis promoting and vessel stabilization functions of BMSCs on ECs, BMSCs have been co-cultured with EPCs or HUVECs in a 3D polyurethane scaffold [37]. Luminal tubular structures were detected after 7 days of culture, which were not only positive for endothelial cell markers CD31 and vWF, but also for CD146, expressed by pericytes but not ECs. Sequential sectioning of scaffolds revealed areas positive for CD146, neuron-glial antigen 2 (NG2), and α-SMA, which were tightly associated with tubular structures. The combination of CD146, NG2, and α-SMA confirmed the pericyte phenotype. Furthermore, neither EPCs nor BMSCs cultured alone could form pre-vascular structures which showed the major role of BMSCs in promoting EPCs to differentiate into a mature network. These experiments further indicated that both cell types influence each other in terms of differentiation. BMSCs did prevent a decrease in number of HUVECs and EPCs that was observed in these cells after 7 days in 3D culture when cultured alone. Interestingly, the study revealed that microvessel-like structures only developed when dexamethasone was added to culture medium. Dexamethasone is known to induce an osteogenic phenotype in BMSCs, which will alter growth factor and extracellular matrix (ECM) protein release by these cells. However, the underlying molecular interactions and alterations during this development remained unclear [37]. Another study investigated the expression of different angiogenic factors (FGF-2, transforming growth factor beta 1 [ TGFB1] and vascular endothelial growth factor A [ VEGFA]) and osteogenic marker genes (collagen type 1 alpha 1 [COL1A1], integrin-binding sialoprotein [IBSP], and transcription factor 7 [SP7]) of BMSCs via qPCR after 7 days in different culture media. While cells cultured in osteogenic medium supplemented with dexamethasone showed enhanced expression of osteogenic marker genes, they showed a significant lower expression of angiogenic genes compared to BMSCs cultured with growth medium free of dexamethasone. These data were further supplemented by an experimental setup using endothelial colony forming cells (ECFCs) and BMSC-conditioned media. While BMSC-conditioned growth medium increased angiogenic stimulation, the addition of dexamethasone showed minimal angiogenic potential [42]. Furthermore, not only culture medium but also matrix composition and cell-cell ratios altered the potential of ECs co-cultured with BMSCs to form tubular networks. Using a collagen Type I 3D matrix for co-culturing HUVECs with BMSCs resulted in a decreased vasculogenic response compared to fibrin-containing matrixes [38]. On the other hand, the ratio between ECs to BMSCs was not a strong modulator of the network development. However, a high EC:BMSC ratio (5:1) showed unstable vessel formation compared to lower ratios [38]. BMSCs did not only promote outgrowth of ECs but also showed a stabilizing function of new vessels, by acting as mural cells and controlling permeability. In contrast to a 3D co-culture model of HUVECs with fibroblasts, HUVECs co-cultured with BMSCs in the same 3D model showed a higher control in permeability due to a higher number of cell-cell adherent junctions between ECs. Vessels supported by BMSCs also showed slower promotion of vessel formation, leading to a higher rate of branches and therefore shorter vessels, which are features indicating physiological vessel growth [43]. While BMSCs provided angiogenic factors and stabilized vascular networks in vitro, they could also act as osteogenic progenitors, forming mineralized bone matrix, thus providing an approach for bone tissue engineering (Fig. 2). Temporal variation of the medium can induce not only the development of vascular networks but also osteogenic differentiation in one and the same co-culture system of HUVECs and BMSCs in vitro, suggesting that BMSCs act as pericytes; the BMSCs even maintained their ability to undergo osteogenesis [44]. To analyze the intercellular communication between BMSCs and ECs, global gene expression was studied. BMSCs and HUVECs were co-cultured for 5 and 15 days to observe relative alterations to the BMSCs previous to co-culture with HUVECs. In this direct culture model, genes related to angiogenesis (vWF, CD31, VE-cadherin, angiopoietin-related protein 4, and CD34) were upregulated. Additionally, the results indicated that the effects of ECs on the BMSC genotype were more prominent after 5 days of co-culture than after 15 days. Among the most upregulated genes were also genes associated with cell adhesion (e.g., CD31 and CD34) and cell-ECM communication (e.g., CD93, cadherin 5 [CDH5], and vWF) suggesting that these processes are also important for crosstalk between BMSCs and ECs. The information upon which genes in BMSCs are upregulated when co-cultured with ECs provided insights about the bidirectional gene regulation of angiogenesis and osteogenesis [45]. Another important regulator of vessel formation is α6β1 integrin, a laminin receptor present on BMSCs. To evaluate the impact of this receptor on EC outgrowth, HUVECs and BMSCs were co-cultured in a 3D fibrin matrix and RNA interference was used to knock down the β6-integrin subunit of the laminin receptor, resulting in 80 % shorter vascular networks in terms of total length compared to HUVECs co-cultured with wild type BMSCs. Furthermore, laminin expression was reduced as well as proliferation of BMSCs in co-cultures in which the RNA interference was induced. Co-cultures of ECs with BMSCs showed no expression of α-SMA in contrast to the control group. This results lead to the suggestion that BMSCs might need the α6β1 to differentiate into pericytes and to induce EC capillary tube formation [46]. Platelet-derived growth factor receptor (PDGFR) is another molecule which showed to be potentially important for angiogenesis. It has been tested if PDGFR signaling influences the migration of BMSCs towards ECs [47]. A Transwell migration assay revealed that HUVECs significantly increased the number of BMSCs migrating across the transwell membrane compared to control medium only. To test whether PDGFR plays a role in this mechanism, imatinib mesylate, an inhibitor of PDGFR, was added to the cell culture medium. This resulted in less migration of BMSCs towards HUVECs in a dose-dependent manner, confirming that PDGFR is at least partly responsible for migration of BMSCs towards ECs [48]. Regarding the formation of tubular networks, studies have been undertaken using a 3D fibrin model in which BMSCs were co-cultured with ECs to investigate the molecular mechanisms of ECM degradation. Results showed that co-culturing with BMSCs lead to an increase in the transcript expression of extracellular proteases in HUVECs. After 3 days of co-culture, capillary structures had formed and RT-PCR revealed enhanced expression of MMPs in ECs. A broad spectrum of MMP inhibitors blocked the capillary network formation in the HUVEC–BMSC co-culture, in contrast to co-cultures of HUVEC with fibroblasts where pre-vascular structures could be observed even when these inhibitors had been added. This indicated that different cells used in co-cultures with ECs also induce different proteolytic enzymes [39]. Based on these findings, further studies in a fibrin based co-culture model were done exploring the specific proteinase dependency in BMSC-mediated angiogenesis [49]. First, the importance of soluble MMPs (MMP-2 and MMP-9) was investigated. Retroviral shRNAs were used to attenuate their expression in ECs. These ECs were then co-cultured with BMSCs, which resulted in unaffected vessel sprouting, compared to co-cultures with mock-infected ECs. As a control, an MMP inhibitor (GM6001) was used, which resulted in drastic inhibition of vessel sprouting. Furthermore, knockdown of membrane type-1 matrix metalloproteinase (MT1-MMP) lead to limited vessel formation in HUVECs. The short vessel-like structures displayed abnormally enlarged lumens. These results suggest that remodeling of the ECM, which is important for invasion of pre-vascular structures, involves MMPs [49].

### Co-cultures of endothelial cells (ECs) with adipose stem cells (ASCs) 

**Fig. 3:**
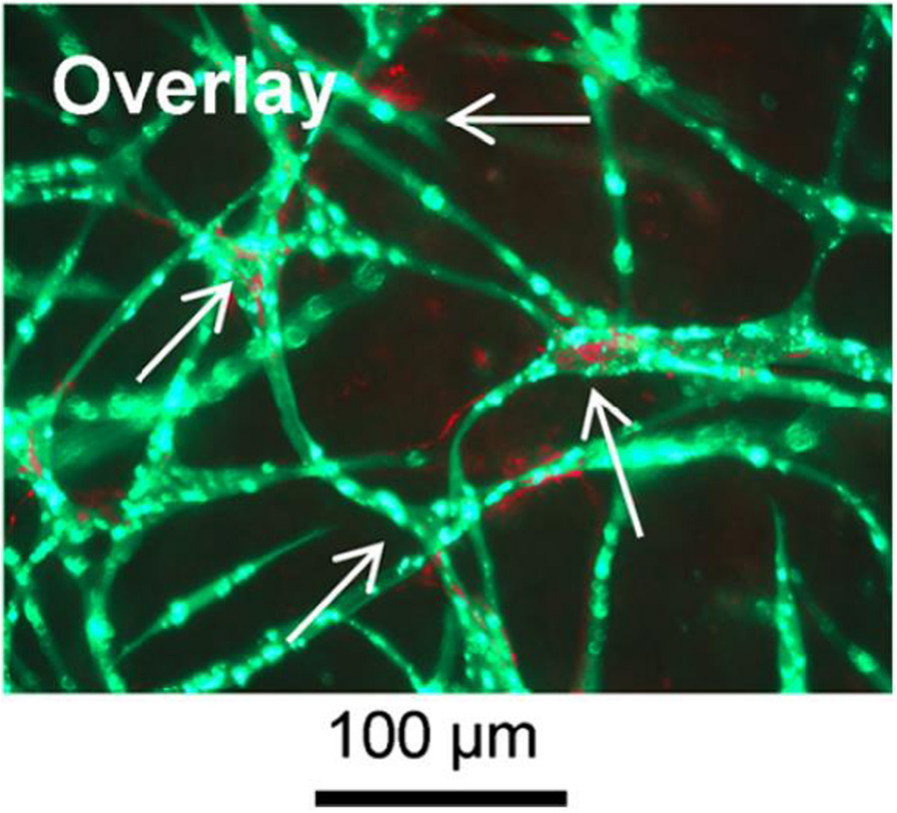
HUVECs (*green*) form pre-vascular structures in co-culture with ASCs (*red*) in fibrin after 4 weeks. HUVECs were transfected with green fluorescence protein to make vascular structures visible under a fluorescence microscope. A staining against NG2 was performed, showing that only ASCs express this surface marker protein. Additionally, it shows that ASCs surrounded the cord-like structures formed by HUVECs(shown by arrows) [33]

Molecular interactions between ASCs and ECs are of interest because there is evidence that ASCs co-cultured with ECs in vitro support the formation of pre-vascular structures and allow network formation (Fig. 3) [9, 33, 43, 50–52]. ASCs participating in the network expressed abundantly α-SMA and CD34 and the cells expressing these molecules surrounded the ECs, suggesting a stabilizing role [9]. Recent data on the molecular mechanisms by which ASCs support angiogenesis and provide vasculogenic functions for ECs are often difficult to interpret and compare due to a lack of standardization and data on factors like media composition, cell ratios, matrix and seeding logistics. One aspect concerning the interaction of ECs and ASCs is to what extent the outgrowth of ECs and the network formation depends on the secreted factors compared to cell-cell interactions and reciprocal signaling. Approaches to address the molecular interactions between these two cell types include direct co-culture or culturing ECs in MSC conditioned medium. Different groups investigated the influence of the supernatants of ASCs on EC outgrowth and mature vessel formation. ASC-conditioned medium had a positive effect on HUVECs and OECs outgrowth [9, 33]. However, the ASC-conditioned medium did not lead to fully branched networks, which indicates that direct cell-cell contact or at least proximity of ASCs with ECs is necessary for tube formation [9, 33, 50]. This assumption was further supported by an experimental setup in which ASCs were seeded on microcarrier beads to retain them to specific sites of a fibrin matrix. There, only ECs near the ASC-coated beads started to form networks [33, 35]. However, not only the EC phenotype changed in co-culture but also the phenotype of the ASCs. Some findings suggested that co-cultured ASCs differentiate towards the smooth muscle lineage. This suggestion was due to the upregulation of α-SMA in cells that surrounded the endothelial cord-like structures in co-cultures eventually stabilizing them and thus representing physiological microvasculature [9, 50]. In addition, it was reported that ASCs express NG2 in regions of bifurcation presence in co-culture with ECs, which indicates the development of pericyte characteristics [33, 53]. These findings substantiate the stabilizing function of ASCs. To investigate the functionality of vessels supported by different mural cells, an inverse permeability model using a dextran (65 kDa) was applied [43] which is one possibility to examine vessel permeability next to in vivo approaches using fluorescently conjugated lectins [15]. Dextran was added to the bulk tissue to reveal incomplete cell-cell junctions by entering immature vessels lumens. Two weeks after start of the co-culture, less dextran was inside the lumen than after 3 days, likely because of mural cells constructs, which made direct contact with the capillary sprouts. Comparing fibroblasts with ASCs used as supporting mural cells in co-culture with HUVEC, ASCs seemed to mimic the physiology of healthy vasculature better than fibroblasts as evidenced by more controlled permeability, which might have been a result of an increase in EC-EC adherens junctions [43]. The molecular interactions responsible for this development remain to be clarified. Another aspect concerning angiogenesis is the proteolysis of the ECM, which depends on the cell type co-cultured with ECs. Co-culture models of HUVECs with ASCs with inhibition of different protease-families showed that the morphogenesis of blood vessels relied on the plasmin family of proteases for EC elongation and invasion. MMPs regulated these parameters to a lesser extent. However, luminal diameter and regulation and possibly vessel stabilization was dependent on these proteinases. Co-culturing HUVECs with ASCs resulted in a promotion of using the plasmin system for proteolysis in HUVECs, while the level of MMPs was unaffected [51]. These data combined with the upregulation of the proangiogenic factors HGF and tumor necrosis factor alpha (TNF-α) within these co-culture systems demonstrated the similarities of molecular communication between co-cultured ASCs or fibroblasts and ECs [51]. On the other hand, a study using OECs instead of HUVECs for a co-culture model with ASCs in a fibrin matrix reported the expression of MMP-14 on these ECs [52]. The results were supported by the fact that robust vessels developed even when a low concentration of aprotinin was added to the medium, which is known to prevent premature fibrin degradation by the plasmin system. According to these findings, OECs form vascular networks supported by ASCs in a fibrin matrix. Furthermore, OECs showed potential molecular differences in fibrin degradation compared to HUVECs [52]. In a setting where co-cultures of ASCs with HUVECs in a fibrin clot were compared to co-cultures of ASCs with OECs, OECs were reported to form a mature network earlier than HUVEC, hinting that different ECs show different molecular interactions within co-cultures with ASCs [33]. Another approach to investigate the molecular interactions of ECs and ASCs in co-culture is the evaluation of gene expression levels via RT-qPCR. Experiments showed an increase in CD31 and VE-cadherin after 1 week of co-culture. These molecules are known to be cellular adhesion molecules which help cells in networks to attach to each other. In addition, VEGFR-2, the main receptor for VEGF and vWF, were also increased, in contrast to platelet-derived growth (PDGF) factor and Tie-2. CD31, VE-cadherin, VEGFR-2, and vWF are known to contribute to the prevention of uncontrolled vessel growth. However, PDGF and Tie-2 are mainly involved in vessel stabilization; therefore, their expression levels might have increased after several more weeks of co-culture [33]. An angiogenesis protein array gave further insight into the molecular interactions of ECs and ASCs in co-culture [33]. After 4 days of culture, EC tube formation was already visible; therefore, supernatants were collected on the fourth day and analyzed. Comparing the results of the assay of OEC monocultures which were cultured with supernatant from ASC monocultures to OEC-ASC co-cultures, the co-culture showed more than two-fold increase of several proangiogenic factors: platelet-derived endothelial cell growth factor (PD-ECGF), FGF-2, MMP-9, Ang-2, and pentraxin-3. This leads to the suggestion that a lack of these angiogenic proteins might be the cause for impaired vessel formation. However, some of these cytokines are known to work in an agonist–antagonist interaction, hence further substantiating that the co-culture of ECs with ASCs leads to physiological, controlled vascular network formation [33]. Addressing the ECM production in co-culture systems of ECs and ASCs, different ECM proteins are reported to be upregulated in co-culture. Collagen IV was not only increased in co-culture but was restricted to the surface of vessel forming cord-blood-derived ECs. Additionally, fibronectin was expressed in higher concentrations and also its extracellular accumulation in fibrillary form was higher. Expression of perlecan I, a heparan sulfate proteoglycan, was also increased in co-culture compared to ASC and EC monoculture, showing an increased amount in proximity to EC vessel structures. Laminin showed no increase but accumulated near EC vascular structures, too. Collagen IV, fibronectin, perlecan I, and laminin are components of the basement membrane, which separate connective tissue from endothelial cells and are therefore associated with vessel formation [54]. According to these finding, cell-cell contacts have an impact on ECM production and therefore on vessel maturation [50].

### Differences between bone marrow-mesenchymal stem cells (MSCs) and adipose stem cells (ASCs) in co-culture with endothelial cells (ECs) 

Comparing the molecular interactions of ASCs and BMSCs with ECs is difficult because while ECs are a rather homogenous population, adult stem cells provide very heterogeneous populations. Therefore, not only the results how ASCs influence ECs compared to BMSCs differ, but in both cases, harvesting, culture methods, and donor variations may additionally be a crucial factor. Furthermore, different populations of ECs (e.g., HUVECs and OECs) showed different reactions in co-culture systems with adult stem cells. Only few studies investigated co-cultures of ECs with ASCs and BMSCs in the same experimental setup to check for differences between these two cell types. Nevertheless, investigations into the molecular interactions of these cell types in co-culture are crucial because both, ASCs and BMSCs, stimulate ECs to form vessel-like structures in vitro. Furthermore, both MSC types supported EC networks by expressing a pericyte-like phenotype (expression of α-SMA and NG2), which makes them very attractive for vascularization techniques in tissue engineering [9]. ASCs and BMSCs are both more favorable than fibroblasts because they do mimic a more physiological vessel growth [43]. Another similarity of ASCs and BMSCs is the need of heterotypic cell-cell contacts or at least the close cellular proximity with ECs to induce network formation. While ASC supernatant has been reported to induce at least outgrowth of ECs [9], BMSC-conditioned medium showed no effect on ECs [33]. Furthermore, investigating the gene expression profile gives an opportunity to observe differences in intercellular communication in ASCs compared to BMSCs. While BMSCs represent the state of the art cell type of MSCs in these co-culture systems, ASCs might be preferred over BMSCs in future. ASCs can be harvested with a less invasive technique and are in general present in higher numbers in adipose tissue than BMSCs are in bone marrow [55]. Differences between BMSCs and ASCs in co-culture with ECs are summarized in Table 1.

**Table 1:** Differences between BMSCs and ASCs in co-culture with ECs

	BMSCs	ASCs
3D matrix used for co-culture	Fibrin [9, 38, 39, 42, 43, 49], polyurethane [37], type I collagen [38], fibrin + type I collagen in different ratios [38]	Fibrin [9, 33, 43, 51, 52]
Pericytic marker	α-SMA [9, 37, 46], NG2 [37], CD146 [37]	α-SMA [9, 50], NG2 [33]
Paracrine factors	HGF, TIMP1, TIMP2 [9]	HGF, TNF-α [51], PD-ECGF, FGF-2, MMP-9, Ang-2, pentraxin-3 [33], ECM proteins: collagen IV, fibronectin [50]
Altered gene expression	FGF-2 ↑, TGFB1 ↑, VEGFA ↑ [42], vWF ↑, CD31 ↑, VE-cadherin ↑, angiopoietin-related protein 4 ↑, CD34 ↑, CD93 ↑, CDH5 ↑ [45], MMPs ↑ [39]	CD31 ↑, VE-cadherin ↑, VEGFR-2 ↑, vWF ↑ [33]
Endothelial cell type	HUVECs [9, 37–39, 43, 45, 46, 48, 49],EPCs [37], ECFCs [42]	HUVECs [9, 33, 43, 51], OECs [33, 52]
MMPs important for network formation and ECM degradation	Yes (in contrast to fibroblasts) [39, 49]	Yes [52], to a lesser extent as plasmin family proteases [51]

BMSCs and ASCs have different effects on ECs and show altered behavior concerning pericytic marker expression, paracrine factors, gene expression, and importance of MMPs for network formation. Furthermore, different matrices as well as different EC types were used to investigate effects of MSCs on ECs

### Functionality of engineered microvessels with mesenchymal stem cells (MSCs) in vivo

**Fig. 4:**
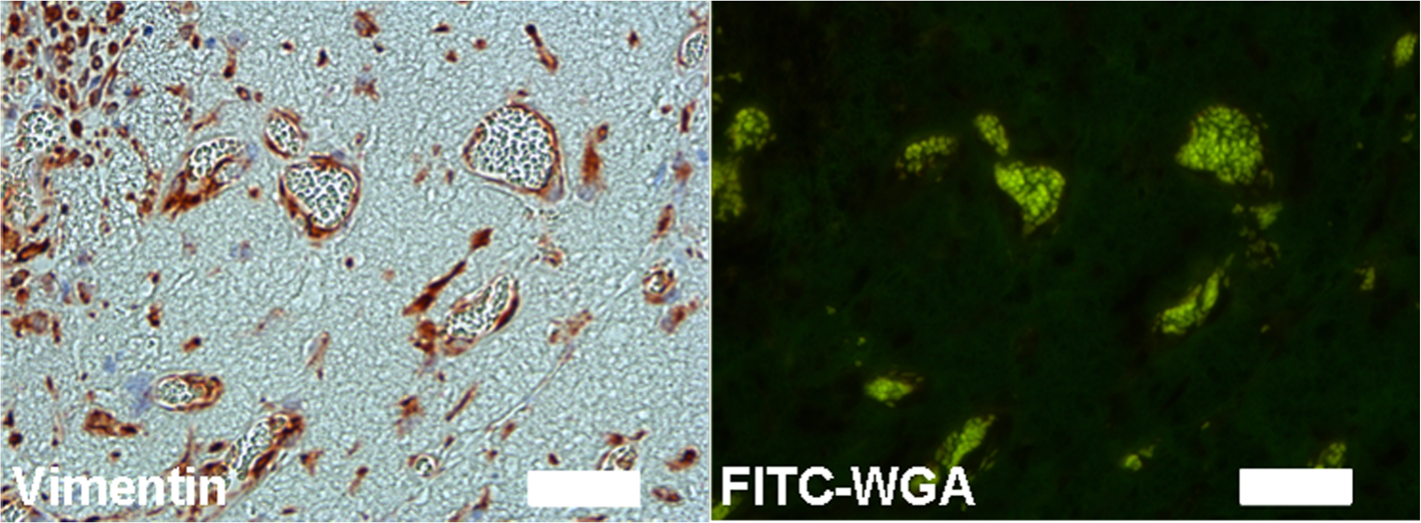
OECs/ASCs co-culture in fibrin subcutaneously implanted in a nude mouse model. ASCs (100,000) were mixed with OECs (100,000) in fibrin (2.5 mg/ml) and pre-vascularized for 1 week in vitro before subcutaneous implantation in a nude mouse. One week later, the clots were excised and processed for immunohistochemistry with anti-human vimentin (*left panel*). Tail-vein-injected FITC-labeled wheat germ agglutinin (WGA) was found in the lumen of these vessels, indicating perfusion and thus functionality (*right panel*). Scale bar = 50 μm

Since analysis of the functionality of microvessel-like structures in vitro is difficult to prove, different in vivo models have been established. EPCs co-cultured with BMSCs show formation of vascular structures in Matrigel as well as in type I collagen, fibrin, and PuraMatrix, an engineered peptide hydrogel, 7 days after the ECM-containing cells were injected subcutaneously in immuno-deficient mice [14, 56]. Such structures could also be found after 2 weeks when a collagen/fibronectin matrix containing EPC-ASC co-cultures was implanted [57]. Even 7 weeks after injection of Matrigel containing ECFC-BMSC co-cultures in nude mice, durable neo-vessel formation was found in Matrigel plugs [58]. A subset of pre-vascular structures formed in in vitro cultured spheroids containing HUVECs as well as ASCs was able to anastomose with the host vascular system of athymic male nude mice [13]. Moreover, co-cultures of OECs and ASCs in fibrin resulted in a perfusable microvascular network when implanted subcutaneously in a nude mouse model (Fig. 4). In most cases, functionality has been verified by locating mouse red blood cells inside vascular structures built by human ECs or by injection of perfusion-indicating agents, for example fluorescently labeled lectins.

## Conclusion

To conclude, ECs and MSCs co-cultured in a 3D matrix provide a promising approach for vascularization in tissue engineering. ASCs and BMSCs are both promising candidates for co-culture systems with ECs because they can be harvested in adults and therefore provide an autologous cell source. While all MSCs seem to have a very similar phenotype, they do interact through different molecular mechanisms with ECs. Co-cultures of BMSCs with various cell types are better researched because they are also used for bone regeneration models, and because there, vascularization remains one of the most limiting factors. On the other hand, the advantage of ASCs is reflected by a less invasive technique, and cells are present in higher numbers. Therefore, it is important to increase efforts whether ASCs or BMSCs provide more promising features for vascularization techniques. Further research in this field will hopefully shed more light on the important signaling mechanisms involved in these co-culture models.

## Abbreviations

3D: three dimensional
α-SMA: alpha smooth muscle actin
Ang: angiopoietin
ASCs: adipose tissue-derived stem/stromal cells
BM: bone marrow
BMSCs: bone marrow-mesenchymal stem cells
CDH5: cadherin 5
COL1A1: collagen type 1 alpha 1
Cx: connexin
ECs: endothelial cells
ECFCs: endothelial colony forming cells
ECM: extra cellular matrix
EGF: epidermal growth factors
EPCs: endothelial progenitor cells
FGF-2: fibroblast growth factor-2
HDMVECs: human dermal microvascular endothelial cells
HGF: hepatocyte growth factor
HUVECs: human umbilical vein endothelial cells
IBSP: integrin-binding sialoprotein
MMPs: matrix metalloproteinases
MSCs: mesenchymal stem cells
MT1-MMP: membrane type-1 matrix metalloproteinase
NG2: neuron-glial antigen 2
OECs: outgrowth endothelial cells
PBMNCs: peripheral blood mononuclear cells
PD-ECGF: platelet-derived epithelial cell growth factor
PDGF: platelet-derived growth factor
PDGFR: platelet-derived growth factor receptor
SP7: transcription factor 7
TGFB1: transforming growth factor beta 1
TIMP: tissue inhibitor of metalloproteinase
TNF-α: tumor necrosis factor alpha
VE-cadherin: vascular endothelial—Cadherin
VEGF: vascular endothelial growth factor
VEGFA: vascular endothelial growth factor A
VEGFR-2: vascular endothelial growth factor receptor—2
vWF: Von Willebrand factor
WGA: wheat germ agglutinin
